# Supplementary material for: Changes in Visual Function and Correlations with Inner Retinal Structure in Acute and Chronic Leber’s Hereditary Optic Neuropathy Patients after Treatment with Idebenone
Source: J Clin Med. 2021 Jan 4;10(1):151. doi: 10.3390/jcm10010151 (PMC7795141; doi:10.3390/jcm10010151)
Supplement: Supplementary file 1 [file jcm-10-00151-s001.pdf]

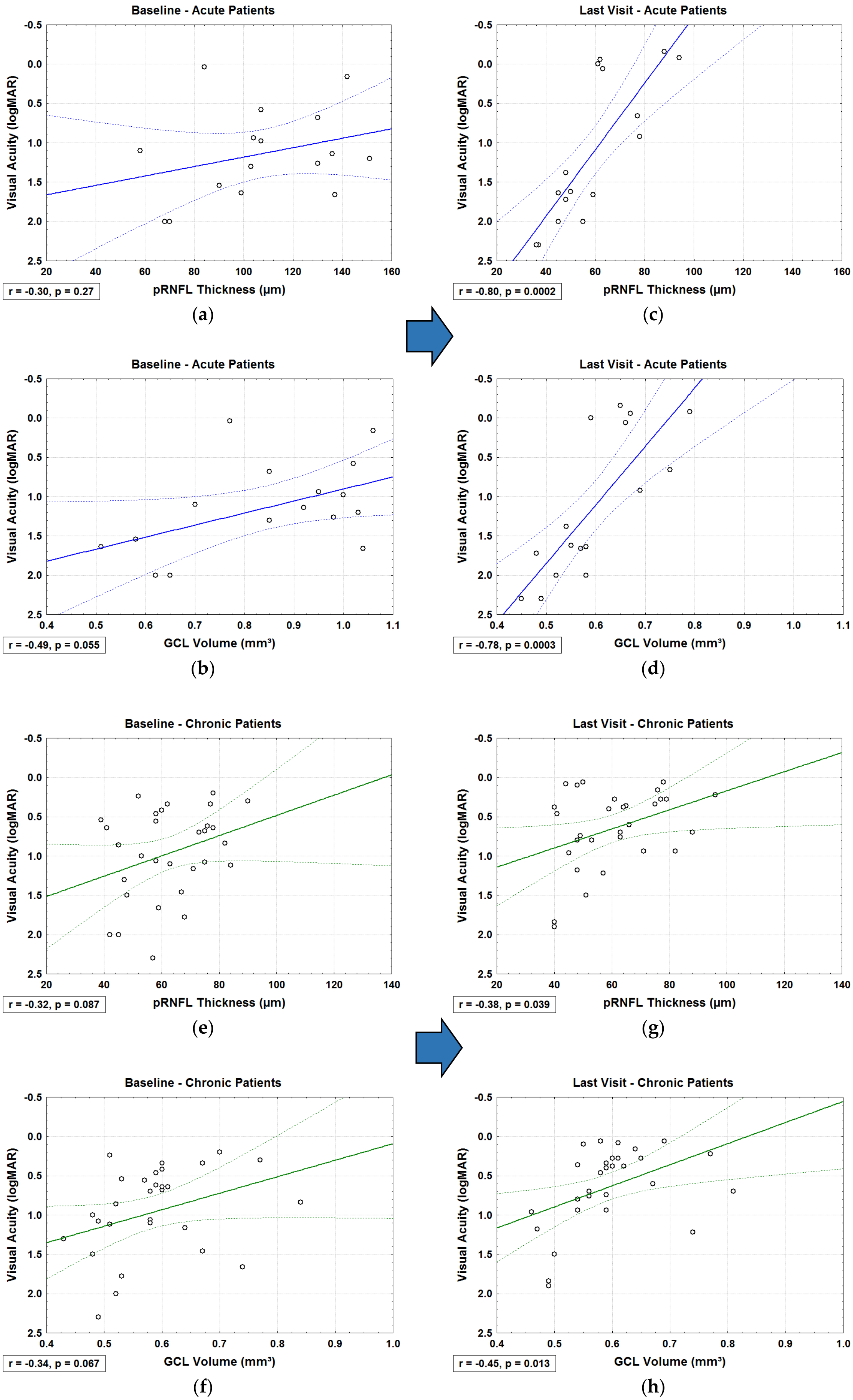

Baseline - Acute Patients
Visual Acuity (logMAR)
pRNFL Thickness (µm)
r = -0.30, p = 0.27
(a)
Last Visit - Acute Patients
Visual Acuity (logMAR)
pRNFL Thickness (µm)
r = -0.80, p = 0.0002
(c)
Baseline - Acute Patients
Visual Acuity (logMAR)
GCL Volume (mm³)
r = -0.49, p = 0.055
(b)
Last Visit - Acute Patients
Visual Acuity (logMAR)
GCL Volume (mm³)
r = -0.78, p = 0.0003
(d)
Baseline - Chronic Patients
Visual Acuity (logMAR)
pRNFL Thickness (µm)
r = -0.32, p = 0.087
(e)
Last Visit - Chronic Patients
Visual Acuity (logMAR)
pRNFL Thickness (µm)
r = -0.38, p = 0.039
(g)
Baseline - Chronic Patients
Visual Acuity (logMAR)
GCL Volume (mm³)
r = -0.34, p = 0.067
(f)
Last Visit - Chronic Patients
Visual Acuity (logMAR)
GCL Volume (mm³)
r = -0.45, p = 0.013
(h)

Figure S1. Scatterplots of correlation analyses between visual acuity and OCT measurements in acute and in chronic LHON patients before and after idebenone treatment. **Legend.** Time from LHON onset at treatment initiation was <1 year in acute patients (a-d) and >1 year in chronic patients (e-h). Significant associations were found only after treatment. (**a**,**e**) Visual acuity (VA) vs. peripapillary retinal nerve fiber layer (pRNFL) thickness at baseline. (**b**,**f**) VA vs. macular ganglion cell layer (GCL) volume at baseline. (**c**,**g**) VA vs. pRNFL thickness at last visit. (**d**,**h**) VA vs. macular GCL volume at last visit. logMAR scales were reversed.

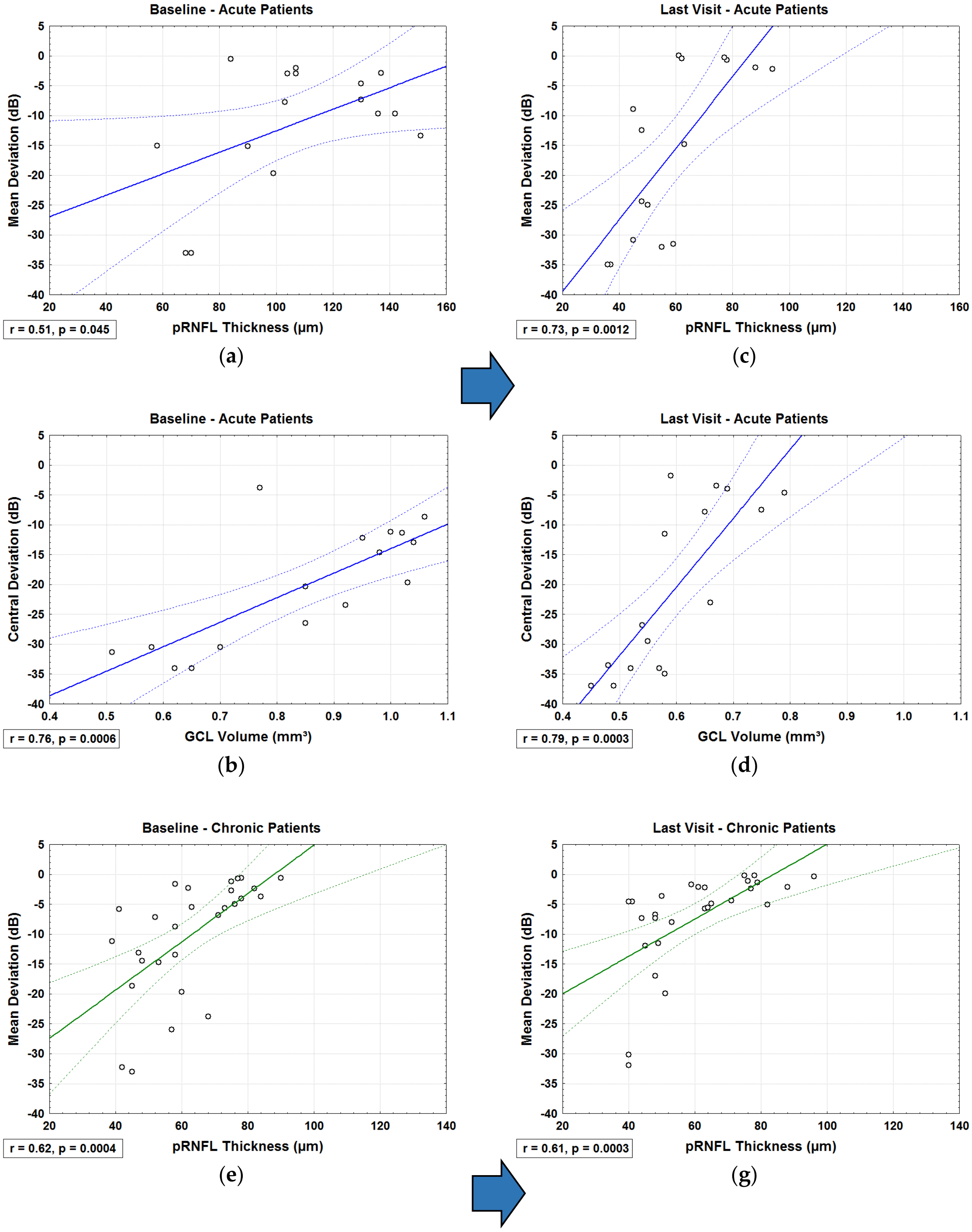

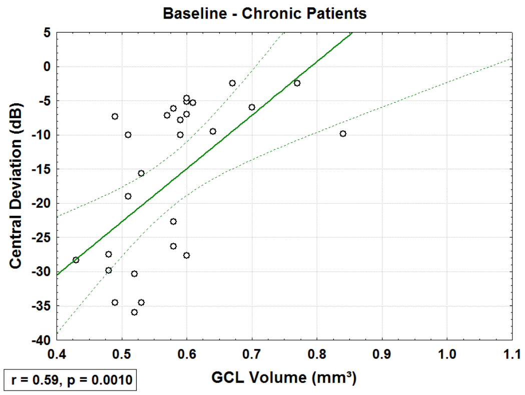

(**f**)

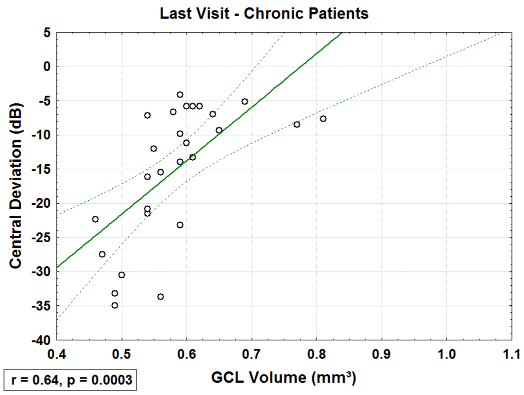

(**h**)

Figure S2. Scatterplots of correlation analyses between visual field and OCT measurements in acute and in chronic LHON patients before and after idebenone treatment. **Legend.** Time from LHON onset at treatment initiation was <1 year in acute patients (a-d) and >1 year in chronic patients (e-h). Stronger associations were found after treatment. (**a**,**e**) Mean deviation (MD) vs. peripapillary retinal nerve fiber layer (pRNFL) thickness at baseline. (**b**,**f**) Central deviation (CD) vs. macular ganglion cell layer (GCL) volume at baseline. (**c**,**g**) MD vs. pRNFL thickness at last visit. (**d**,**h**) CD vs. macular GCL volume at last visit.
